# Genome-wide association studies in lettuce reveal the interplay of seed age, color, and germination under high temperatures

**DOI:** 10.1038/s41598-024-84197-3

**Published:** 2025-01-03

**Authors:** Sookyung Oh, Ezekiel Ahn, Ainong Shi, Beiquan Mou, Sunchung Park

**Affiliations:** 1 U.S. Department of Agriculture, Agricultural Research Service, Beltsville, MD 20705 USA; 2 Horticulture Department, University of Arkansas, Fayetteville, AR 72701 USA; 3 U.S. Department of Agriculture, Agricultural Research Service, Salinas, CA 93905 USA

**Keywords:** Lactuca, Lettuce, Thermoinhibition, Seed color, Seed age, GWAS, Agricultural genetics, Plant genetics, Quantitative trait, Genetic association study, Genome-wide association studies, Plant genetics, Plant sciences, Plant stress responses, Heat

## Abstract

Thermoinhibition, the suppression of seed germination by high temperatures, is an adaptive trait that ensures successful seedling establishment in natural environments. While beneficial for wild plants, thermoinhibition can adversely affect crop yields due to uneven and reduced germination rates, particularly in the face of climate change. To understand the genetic basis of thermoinhibition, we conducted a comprehensive genetic analysis of a diverse panel of *Lactuca* spp. accessions. Our findings revealed that *L. serriola*, a wild ancestor of cultivated lettuce, showed relaxed thermoinhibition response compared to cultivated lettuce, suggesting that this trait may have been positively selected during early domestication in the Mediterranean region with hot, dry summers. Additionally, we found that thermoinhibition intensified with seed age but was less pronounced in dark-colored seeds, which showed increased germination under high temperatures. Genome-wide association studies identified genomic regions associated with thermoinhibition, including genes involved in ethylene and ABA signaling. Interestingly, some of these regions were also linked to seed color, suggesting a potential genetic coupling between black seed color and reduced thermoinhibition. These results highlighted the complex interplay between thermoinhibition, seed color, and domestication in lettuce, indicating a complicated nature of thermoinhibition regulation. By elucidating the genetic architecture of thermoinhibition, our study provides a valuable foundation for breeding strategies to enhance lettuce resilience to climate change.

**Supplementary Information:**

The online version contains supplementary material available at 10.1038/s41598-024-84197-3.

## Introduction

Seed dormancy and thermoinhibition are two pivotal physiological mechanisms that regulate the timing of seed germination, ensuring that seedling emergence aligns with favorable environmental conditions. While both processes serve to optimize the survival and establishment of seedlings, they are distinct in their environmental triggers and ecological implications. Seed dormancy is an adaptive mechanism that prevents premature germination of viable seeds, even under favorable conditions that might be transient. This mechanism is critical for synchronizing germination with optimal environmental cues, primarily temperature changes that signal the appropriate season for growth^1,2^.

In contrast to seed dormancy, seed thermoinhibition is a response mechanism that temporarily suspends germination during periods of excessively high temperatures^3,4^. Unlike dormancy, which is a more static state, thermoinhibition can be quickly reversed upon the return to favorable temperature conditions. This process is exemplified by lettuce seeds, which exhibit minimal primary dormancy but are highly susceptible to thermoinhibition^3,4^. Originating from the Mediterranean region^5^—a climate characterized by hot, dry summers—lettuce seeds may have evolved thermoinhibition as a protective adaptation to delay germination until the decline of summer heat, thereby aligning seedling emergence with cooler, moist conditions conducive to growth^6,7^.

While thermoinhibition may confer advantages in natural settings, it poses significant challenges in agriculture, particularly as global temperatures continue to rise. The increasing prevalence of heat waves and altered thermal profiles due to climate change threaten to disrupt germination uniformity, leading to reduced agricultural yields^8,9^.

The upper temperature threshold for germination varies significantly across lettuce genotypes^10–13^, reflecting a broad spectrum of ecological adaptations. Most cultivars, including cv Salinas, fail to germinate at 29 °C or higher temperatures due to strong thermoinhibition. In contrast, certain thermotolerant genotypes exhibit remarkably high germination rates–above 90%–even at even at 36 °C^14^, with *L. serriola*, the wild progenitor of cultivated lettuce, showing greater thermotolerance^11,14^, possibly as a consequence of its adaptation to hotter, drier climate conditions.

Seed priming–prehydrating seeds at low temperatures followed by drying before sowing–has been employed to mitigate thermoinhibition by allowing seeds to initiate but not complete germination processes. This partial hydration enhances seed resilience to high-temperature stresses^15,16^. Despite its effectiveness, seed priming faces several practical challenges on a large scale. Increased moisture levels can lead to seed decay and disease, necessitating strict sanitation protocols that add cost and complexity^17^. Therefore, leveraging natural genetic variations to counter seed thermoinhibition presents a sustainable alternative to enhance crop establishment and productivity in lettuce, helping to combat the impacts of global warming.

Phytohormones play critical roles in seed germination. Abscisic acid (ABA), the primary hormone responsible for inhibiting germination, is crucial in maintaining dormancy^18^. ABA is synthesized during seed development, contributing to storage reserve accumulation and desiccation tolerance and counteracting the effects of gibberellic acid (GA) that promote germination. In-depth genetic studies have identified key genes in thermoinhibition such as *9-CIS-EPOXYCAROTENOID DIOXYGENASE4* (*LsNCED4*), which is essential for ABA synthesis and contributes significantly to the thermotolerance observed in *L. serriola* US96UC23 ^19,20^. Additionally, mutant screening in lettuce has identified genes like *LsABA1*, which negatively influences ABA-mediated germination inhibition. Mutations in *LsABA1* have been shown to reduce thermoinhibition, underscoring the critical role of ABA signaling in seed thermoinhibition^21^.

Ethylene is also known for its significant role in modulating germination and thermoinhibition^22^. Recognized for breaking dormancy in Compositae family members like lettuce and sunflower, ethylene application can alleviate the inhibitory effects of high temperatures on seed germination and reduce overall seed dormancy^3,23^. Ethylene production in lettuce seeds decreases with rising temperatures during water uptake, indicating a temperature-sensitive synthesis pathway that may impact dormancy and thermoinhibition dynamics^24,25^. Moreover, ethylene may enhance germination indirectly through interactions with GA and ABA. Ethylene can enhance GA biosynthesis or activity, which facilitates germination even under higher temperatures. It can also reduce ABA synthesis, increase ABA degradation, or decrease the sensitivity to ABA, effectively modulating the inhibitory role of ABA in seed germination^26–28^. This hormonal interplay was further evidenced by the coordinated expression of genes involved in ethylene and GA synthesis in response to germination-inducing temperatures^12^. Additionally, recent genetic and transcriptomic analyses have reinforced the importance of ethylene signaling in seed germination during high-temperature imbibition. A lettuce homolog of *ETHYLENE RESPONSE FACTOR1*, *LsERF1* that is also known as *LsERF172*^29^, was identified as a causal locus for thermoinhibition in a quantitative trait locus (QTL) analysis^30^.

Gibberellic acid is essential for overcoming seed thermoinhibition by counterbalancing the inhibitory effects of ABA^10,31^. GA enhances seed germination by promoting ABA catabolism or reducing ABA sensitivity. Moreover, GA has been shown to induce the expression of enzymes responsible for ethylene biosynthesis, thus facilitating a hormonal synergy that supports germination. Conversely, inhibiting ethylene synthesis can lead to a decrease in the expression of GA biosynthesis enzymes, demonstrating the tightly linked regulatory network governing seed hormonal responses^1,32^.

Lettuce is a world-wide popular leafy vegetable for its health and nutritional benefits, including dietary fiber, minerals, and vitamins^33^. However, the increasing occurrence of unseasonal and erratic high temperatures associated with climate change poses significant challenges to its cultivation. Unseasonal high temperatures can disrupt the synchronization of germination, leading to decreased and uneven lettuce yields. Therefore, the development of cultivars with enhanced thermotolerant germination has emerged as a crucial objective in lettuce breeding^4^.

Current knowledge of thermoinhibition in lettuce has primarily focused on certain cultivars, with extensive studies on *L. serriola* leading to the identification of several genes involved in this process^30,34^. However, the rich genetic diversity observed among lettuce accessions for thermoinhibition suggested that multiple regulatory mechanisms were involved, highlighting the complexity of this trait^14^. To address this knowledge gap, our study explored the genetic basis of thermoinhibition by analyzing a diverse panel of lettuce accessions through genome-wide association studies (GWAS). This analysis led to the discovery of novel loci associated with thermoinhibition, underscoring the potential presence of various genetic mechanisms regulating this trait. Additionally, our study expanded to examine how seed age and color influence thermoinhibition. These factors were systematically analyzed to understand their impact on germination rates under high temperatures. Our findings revealed a potential interplay of seed color, seed age, and thermoinhibition. This research not only broadens our understanding of the genetic factors governing thermoinhibition but also provides breeders with valuable genetic tools to develop lettuce varieties that are better adapted to the challenges posed by climate change.

## Results

### Seed age effect on thermoinhibition

**Fig. 1:**
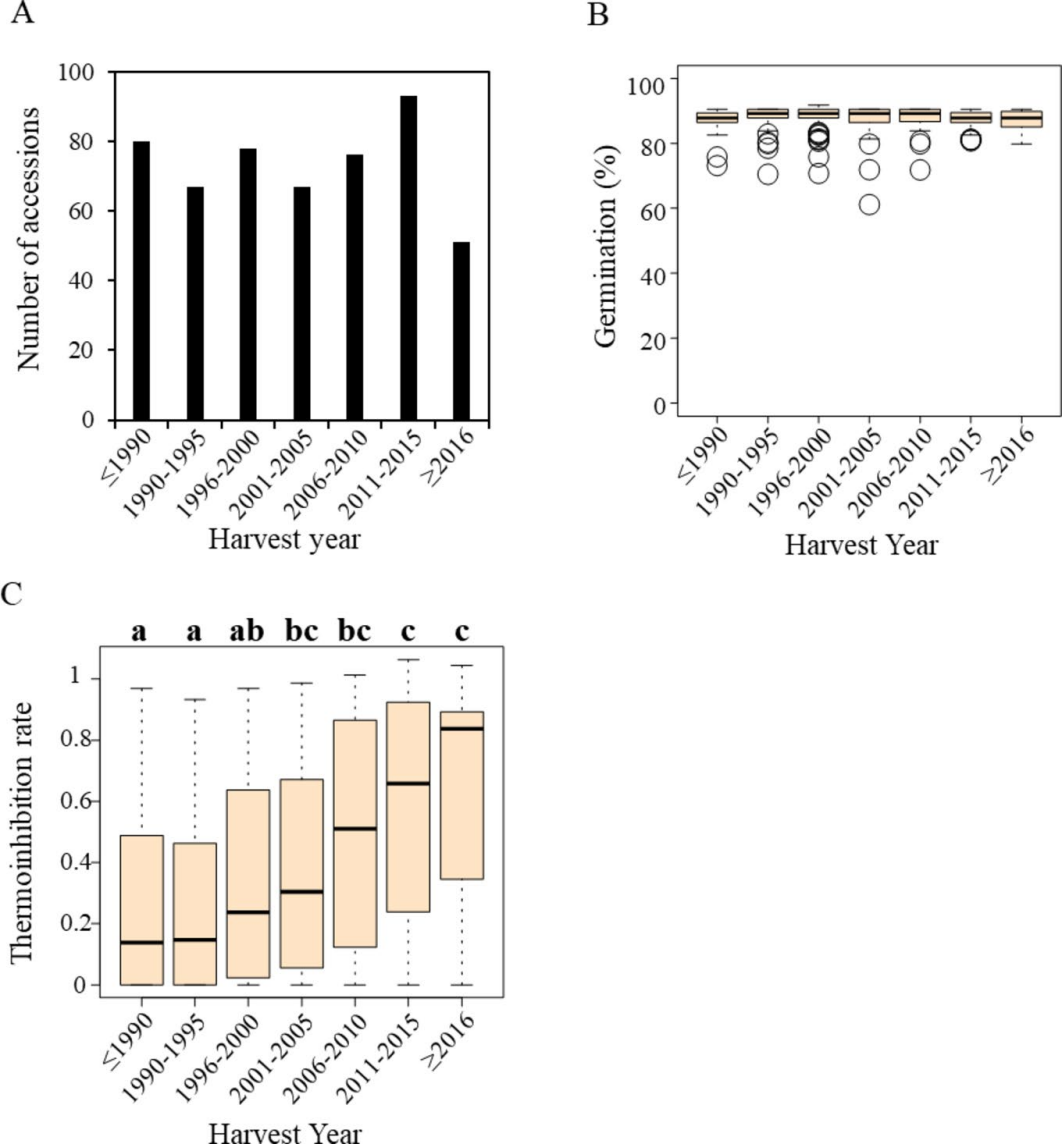
Impact of harvest year on lettuce seed germination under high-temperature conditions (34 °C). (**A**) Distribution of lettuce seeds across different harvest years. (**B**) Germination rates of lettuce seeds from various harvest years under control temperature conditions (21 °C). (**C**) Thermoinhibition rates, calculated as the ratio of germination rates under high-temperature conditions to those under control conditions. Different letters at the top indicate statistically significant differences among groups (Tukey’s HSD test, alpha = 0.01).

The seeds from 521 *Lactuca* spp. accessions (Table S1) were evaluated for germination rate under high-temperature (34 °C) and control (21 °C) conditions, where the seeds were harvested at various years between 1977 and 2017 (Fig. 1A). Under control conditions, the seeds exhibited a high average germination rate of 87.4%. To assess the possible age effect on germination rates, the seeds were grouped into seven groups by harvest year. The harvest groups exhibited similar germination rates under control conditions (Fig. 1B). For example, the seeds harvested from 2006 to 2010 displayed the highest germination rate at **88.2**%, whereas those from 2016 to 2017 showed the lowest at **87.1**%. An ANOVA test confirmed that these differences between harvest years were not statistically significant (P-value = 0.59), suggesting no correlation between germination rate under control conditions and seed harvest year.

However, under high temperature conditions, an inverse correlation was observed between seed age and germination rate. The oldest seed group (harvested in 1990 or before) exhibited the lowest germination rate at **27.7**%, while most recently harvested seeds (years 2016 and 2017) achieved the highest rate at **64.7**% (Fig. 1C). Significance of the variation between harvest year groups was supported by an ANOVA test (P-value = 5.45E-14) (Table S2), indicating a pronounced effect of seed age on thermoinhibition, with thermoinhibition strengthening over time after harvest.

### Heat-tolerant germination of *L. Serriola*

**Fig. 2:**
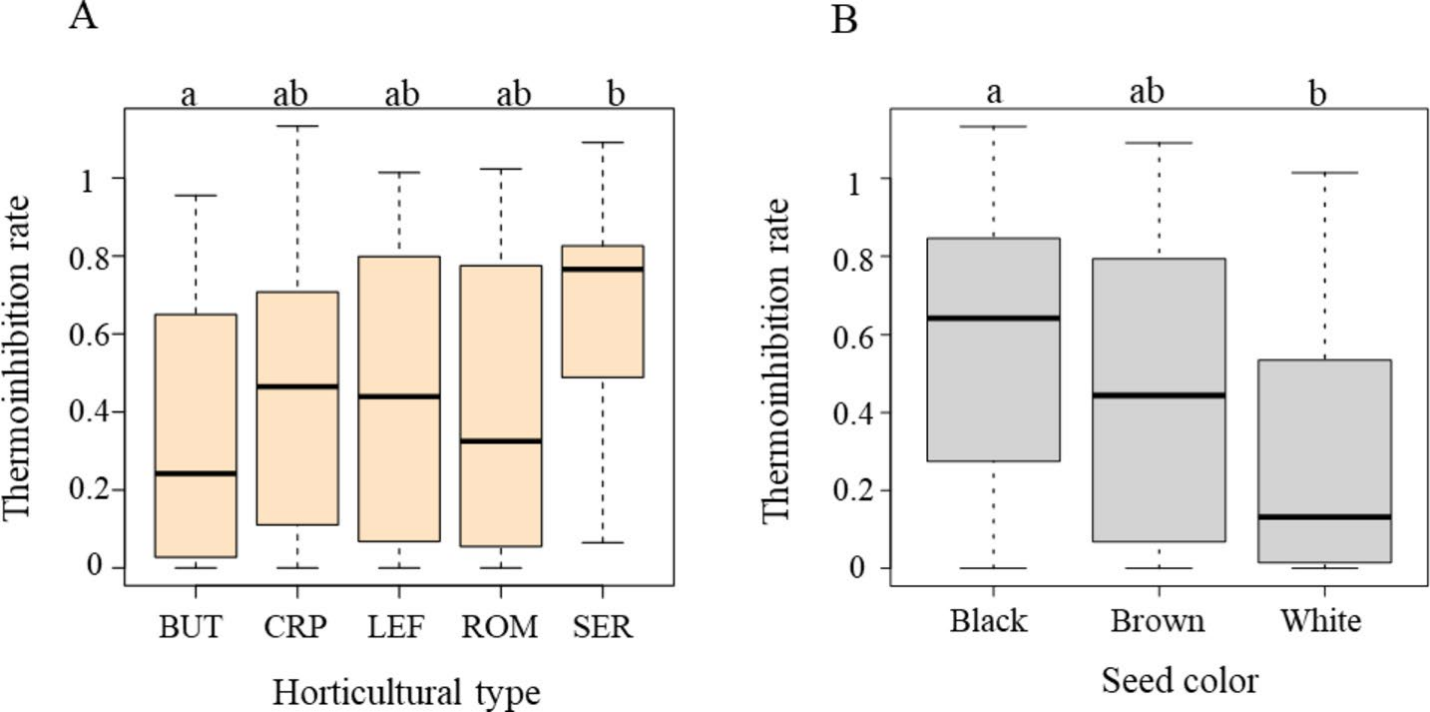
Influence of horticultural types and seed colors on thermoinhibition at 34 °C. (**A**) Comparison of thermoinhibition rates among horticultural types including butterhead (BUT), crisphead (CRP), leaf (LEF), and romaine (ROM), and *L. serriola* (SER). (**B**) Thermoinhibition rate based on seed colors–black, brown, and white–where thermoinhibition rate is represented as the ratio of germination rates under high-temperature conditions relative to those under control conditions. Statistically significant differences are indicated by different letters at the top of the plots (Tukey’s HSD, alpha = 0.01).

To control for seed age variability, we conducted the germination test using freshly harvested seeds from the same accessions. The germination test revealed an average thermoinhibition rate of 41.5% under high-temperature conditions at 34 °C. Among the 521 accessions, 12% (*n* = 62) were unable to germinate at 34 °C. The remaining accessions displayed a wide range of thermoinhibition rates: 1–25% (164 lines), 26–50% (68 lines), 51–75% (105 lines), and 76–100% (115 lines), while seven lines exhibited slightly higher germination rate under high temperatures compared to control temperatures (Fig. S1, Table S1). When thermoinhibition rate was evaluated for four different horticultural types of cultivated lettuce and wild lettuce (*L. serriola*), the wild lettuce showed less pronounced thermoinhibition with a rate of 63%, while cultivated lettuce types showed an average of 40.7% (33% for butterhead, 41% for romaine, and 44% for crisphead and leaf type) (Fig. 2A; Table S1). The significance of these differences was confirmed by an ANOVA test (P-value = 0.014) (Table S3), and Tukey’s HSD test (alpha = 0.05) supported significant difference between *L. serriola* and butterhead type (Fig. 2A).

### Thermoinhibition variation by seed color

Seeds from the GWAS panel displayed different colors, divided into three groups: 237 accessions for black, 17 for brown, and 259 for white. To assess an association between seed color and thermoinhibition, we examined germination rate among seed color groups. Seeds with a black pericarp exhibited the highest germination rate under heat conditions of 55.4%, followed by brown at 44% and white at 27.8% (Fig. 2B). The significance of this difference across seed colors was confirmed by the ANOVA test (P-value = 2E-16) (Table S4) and further substantiated by the Tukey’s HSD test, particularly noting the significant disparity between black and white seeds (Fig. 2B). These results indicated that germination inhibition by heat (thermoinhibition effect) was attenuated in darker-colored seeds compared to white-colored seeds.

### Genome-wide association study on seed thermoinhibition

**Fig. 3:**
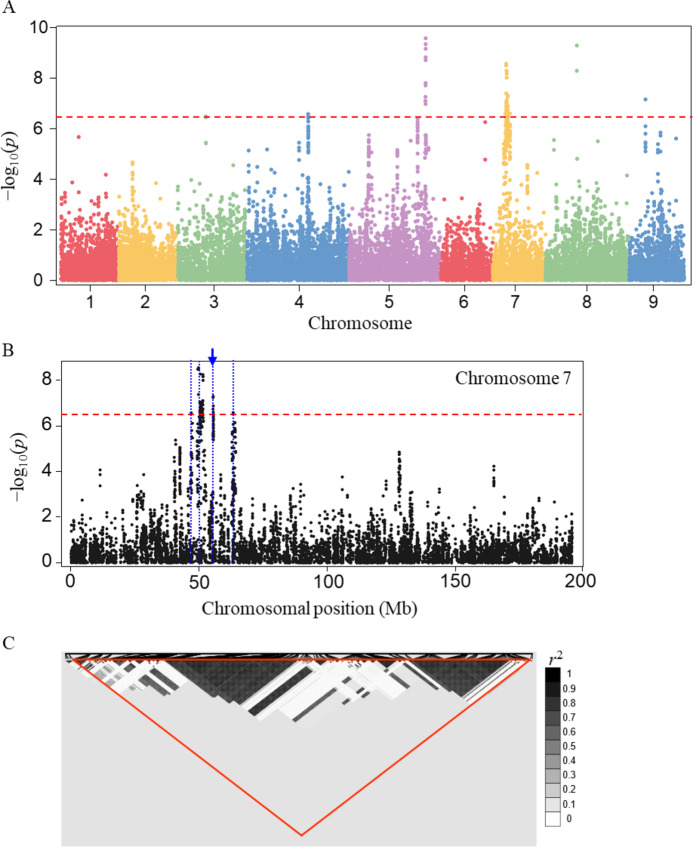
Analysis of SNPs associated with thermoinhibition. (**A**) Manhattan plot illustrating significant SNPs associated with thermoinhibition across the genome. The red horizontal dashed line represents the genome-wide significance threshold (P-value < 2.7E-7, −log_10_(*p) =* 6.57). (**B**) Detailed Manhattan plot for chromosome 7 showing significant SNPs. The blue vertical dashed line indicates the positions of candidate genomic regions identified by LD block analysis. (**C**) Visualization of the largest LD block (indicated by a blue arrow in panel B), with LD strength expressed as *r*^2^.

To explore the genetic basis of variation in thermoinhibition, we conducted a GWAS using SNP data from various *Lactuca* spp. accessions. We identified 84 SNPs significantly associated with thermoinhibition, with a cutoff of Bonferroni-corrected P-value of 2.7E-7 (Table S5). These SNPs were distributed across five chromosomes including 4, 5, 7, 8, and 9 (Fig. 3A). To identify potential candidate genes, we determined linkage disequilibrium (LD) blocks where the significant SNPs were linked to their neighboring SNPs by an LD of *r*^2^ > 0.5. The blocks spanned a total length of 4.6 Mb in the genome with an average size of approximately 0.52 Mb (Table S6). Chromosome 4, 8, and 9 each contained a single LD block, while chromosome 5 contained two and chromosome 7 contained four blocks, among which the largest block spanned 2.3 Mb on chromosome 7 (Fig. 3B and C). Within the blocks, a total of 91 genes were identified based on the lettuce reference genome annotation (version 8) (Table S7).

Further annotation of these 91 genes using Gene Ontology (GO) biological process highlighted their potential biological functions. We particularly focused on genes associated with heat, seed germination, or phytohormone-signaling as phytohormones, including ABA, ethylene, and GA, are known to play important roles in seed germination. Among the 91 genes, six genes were associated with the focused functions (Table 1). A gene associated with a GO function of ‘response to heat’ (GO:0009408) encoded a chaperone protein, similar to Gametophytic factor 2 (GFA2, At5g48030) of *Arabidopsis*, which has been known for its responsiveness to heat stress^35^ and its involvement in female gamete development^36^. A gene encoding GATA transcription factor 22 was associated with ‘response to gibberellin’ (GO:0010029) and ‘regulation of seed germination’ (GO:0009739). Additionally, two genes were associated with ethylene signaling: one gene encoded a protein closely related to ENHANCED ETHYLENE RESPONSE PROTEIN 5 (EER5, AT2G19560) of *Arabidopsis*, whose mutation showed ethylene hypersensitivity in *Arabidopsis*^37^ and the other gene encoded an enzyme, 1-aminocyclopropane-1-carboxylate oxidase 1 (ACO1, At2g19590), which is involved in the final step of ethylene biosynthesis in plants^38^. ACO1 has been previously reported to enhance seed germination under heat stress conditions^39^. Two genes were identified to be associated with abscisic acid signaling: one gene was closely related to ABA INCENTIVE 1 (ABI1, At4g26080), a negative regulator of ABA signaling^40^ and the other gene encoding Calcium-dependent protein kinase 13 was known for its involvement in ABA-signaling^41^.

**Table 1:**
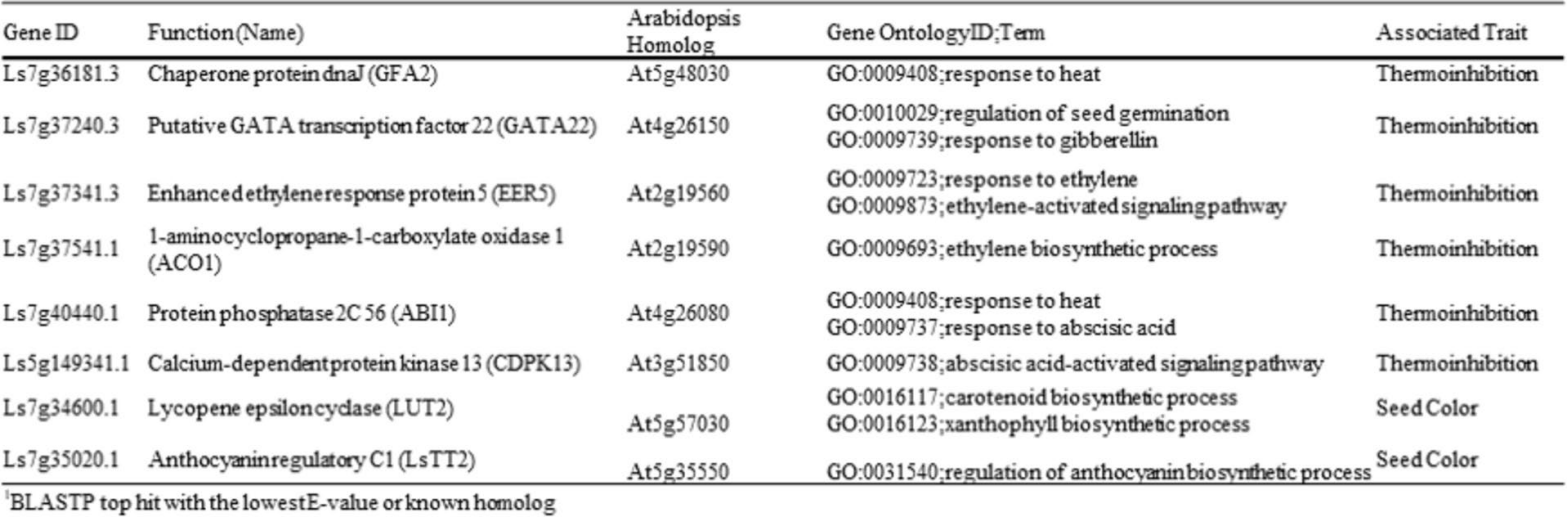
Candidate genes associated with thermoinhibition and seed color, and their functional annotations.

### Association between seed color and thermoinhibition

Given the significant difference in thermoinhibition rate among seeds of different colors (Fig. 2B), we examined whether genes influencing seed color might be associated with thermoinhibition. The GWAS population had 211 accessions with black seeds, 235 with white seeds, and 14 with brown seeds, and we conducted a GWAS to investigate the genetic basis of the seed color variation.

**Fig. 4:**
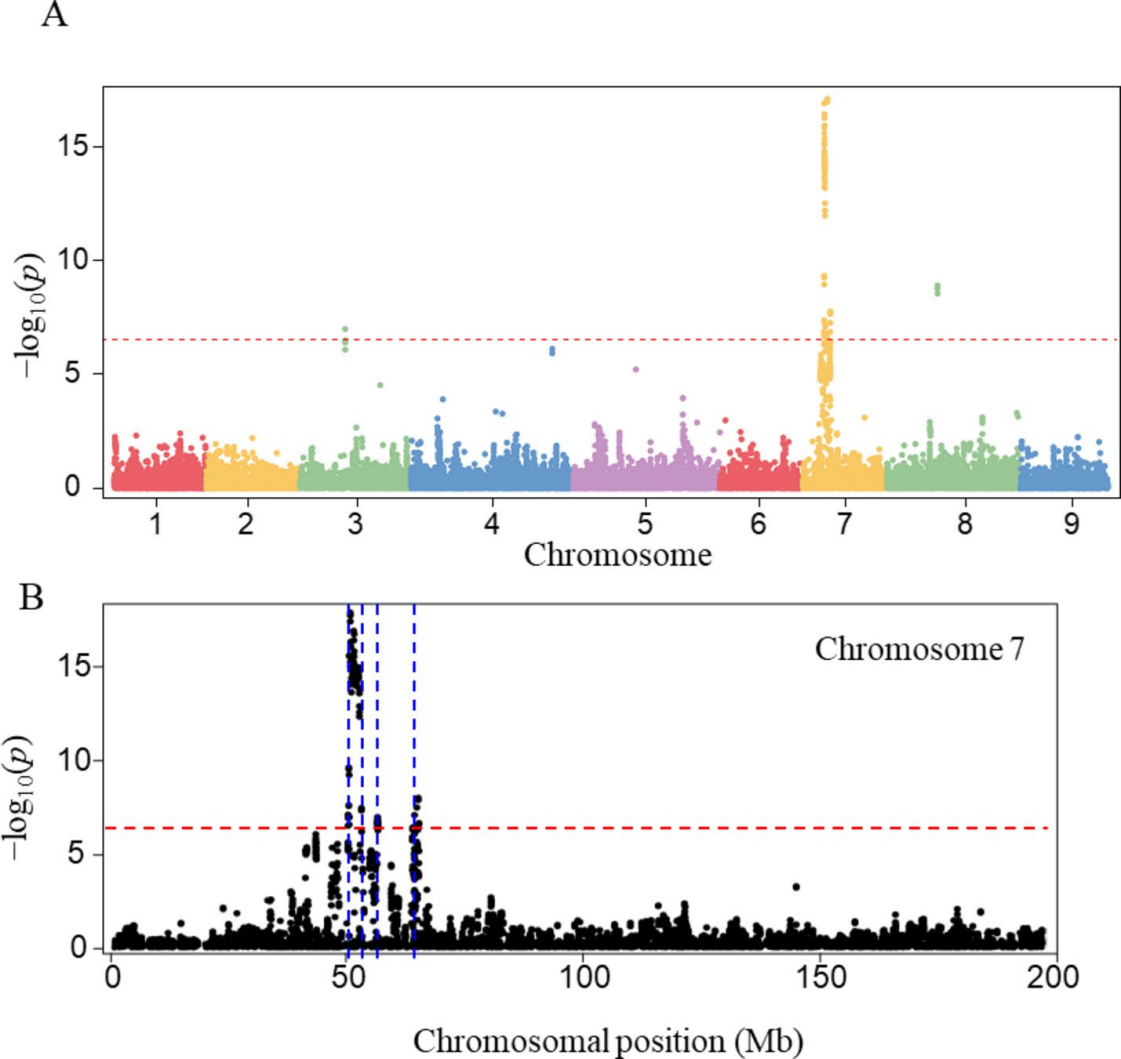
Manhattan plot analysis for seed color. (**A**) Manhattan plot showing significant SNPs associated with seed color across the genome. (**B**) Manhattan plot for chromosome 7. The red horizontal dashed lines indicate the genome-wide significance threshold (P-value < 2.7E-7, −log_10_(*p) =* 6.57). The blue vertical dashed lines indicate the positions of candidate genomic regions identified by LD block analysis.

By applying the same significance threshold as for thermoinhibition, we identified 211 SNPs significantly associated with seed color variations (Fig. 4A, Table S8). Most of these SNPs (*n* = 206) were located on chromosome 7, while one SNP was located on chromosome 3 and four SNPs were located on chromosome 8.

Subsequent pairwise LD analysis resulted in six LD blocks, totaling 4.7 Mb. Four candidate LD blocks were located on chromosome 7, with sizes of 0.26 Mb, 0.35 Mb, 1.1 Mb, and 2.6 Mb, respectively (Fig. 4B, Table S6). Chromosome 3 and 8 each contained a single block of 0.2 Mb (Table S6). These six blocks contained 91 genes (Table S9). Among these, there were two genes associated with pigment biosynthesis (Table 1). One gene encoded an enzyme, Lycopene epsilon cyclase, known for its involvement in carotenoid biosynthesis^42,43^. The other gene encoded anthocyanin regulatory C1, a MYB transcription factor, previously recognized as a causal locus (*LsTT2*) for lettuce seed color in a GWAS analysis^44^, where the authors showed that a stop codon point mutation of this gene resulted in white-colored seeds. The identification of this gene in our study reinforced the validity of our results.

**Fig. 5:**
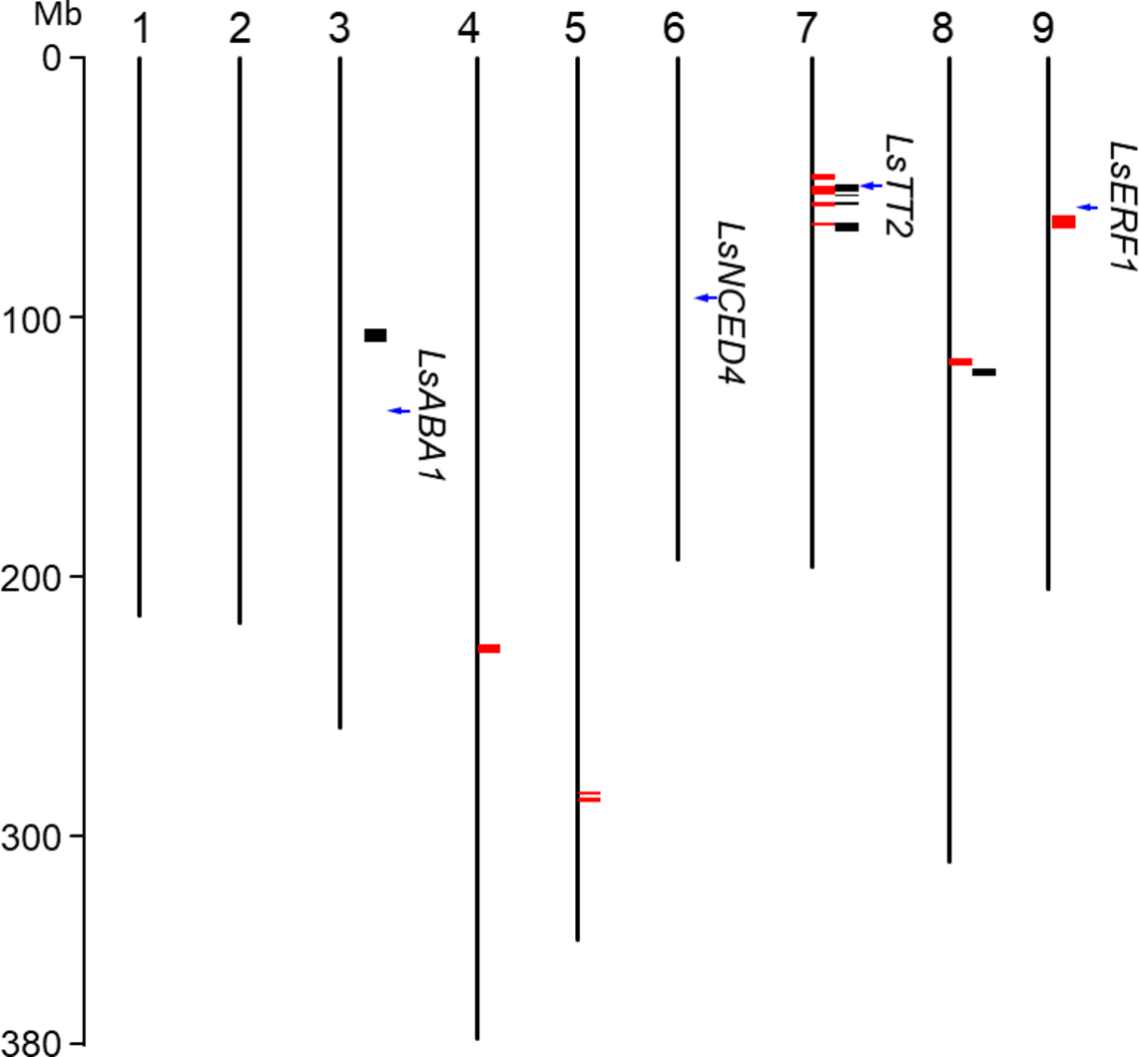
Genomic distribution of QTNs and causal loci associated with thermoinhibition and seed color. Thermoinhibition QTNs (red bars) were compared with seed color QTNs (black bars). The co-localization of three QTN blocks on chromosome 7 suggested a potential genetic link between the two traits. Previously identified causal loci for thermoinhibition (*LsERF1*, *LsNCED4*, and *LsABA1*) and seed color (*LsTT2*) were indicated by blue arrows. The scale bar on the left indicates a chromosomal position in megabases (Mb).

Interestingly, three of the seed color LD blocks on chromosome 7 colocalized with the candidate blocks of thermoinhibition trait (Fig. 5). Specifically, 54 of the total 91 candidate genes on chromosome 7 were shared between seed color and thermoinhibition traits (Table S9), including the four key candidates, *GATA22*, *EER5*, *ACO1*, and *ABI1*. This result suggested a potentially shared genetic pathway influencing both seed color and thermoinhibition.

## Discussion

Seed thermoinhibition, a natural adaptation that prevents germination during periods of heat stress, can hinder agricultural production, where consistent and timely germination is crucial regardless of temperature fluctuations^45,46^. As climate change exacerbates temperature variability with rising temperatures and more frequent heat waves, reducing thermoinhibition is crucial for ensuring crop resilience and maintaining consistent germination across a broader temperature range. In this study, we investigated the genetic mechanisms underlying seed thermoinhibition in lettuce, an economically important vegetable crop.

An intriguing observation from our study is that aged seeds exhibited stronger thermoinhibition (i.e., reduced germination rates under high temperatures) compared to freshly harvested seeds, suggesting that over time, lettuce seeds may enhance their thermoinhibitory responses. Prolonged seed storage often reduces seed viability and longevity, most likely due to accumulated oxidative damage to cell macromolecules such as proteins, sugars, lipids, and nucleic acids^47–49^. However, our findings indicated that the increased thermoinhibition was not attributable to reduced viability, as seeds of various ages showed comparable germination rates under control conditions (ANOVA test P-value = 0.59). Notably, the oldest seeds in our study, harvested as far back as 1977, maintained a high germination rate of nearly 85% at the control temperature of 21 °C. This age-related increase in thermoinhibition underscores the dynamic nature of the mechanisms underlying thermoinhibition, which may involve complex physiological changes occurring during long-term storage of seeds at − 20 °C, the storage condition for USDA lettuce germplasm collections.

One potential physiological change could be the accumulation of germination-inhibitory hormones, such as ABA, which plays a crucial role in inhibiting seed germination. Storing seeds at − 20 °C, a condition that simulates freezing and drought stresses, could promote ABA signaling^50,51^, leading to enhanced thermoinhibition.

Alternatively, physical changes in the seed structure, like increased rigidity of the endosperm, might also enhance the thermoinhibitory effect^4,52^. The endosperm plays a multifaceted role, acting as a physical barrier that regulates embryo growth and development while also providing nutrients and protection^53,54^. Studies have shown that gradual hardening of the endosperm occurred during storage, which had a strong correlation with thermoinhibition^55^.

To fully understand the mechanisms underlying thermoinhibition increase in aged seeds, further studies should focus on the biochemical and structural changes in seeds over time, particularly in those accessions where seed viability and low thermoinhibition rates were maintained during storage at − 20 °C. Such studies could provide strategies for agricultural practices and seed storage aimed at optimizing germination and crop yields under varying climatic conditions.

Our study highlighted a colocalization of casual loci for seed color and thermoinhibition on chromosome 7 (Fig. 5), where three candidate LD blocks overlapped between these traits, sharing 56 candidate genes. This observation suggests two possible scenarios: (1) the traits are tightly linked due to the physical proximity of their associated genes on the chromosome 7, or (2) they share a common regulatory pathway, indicating pleiotropic effects.

Previous research identified *LsTT2*, an R2R3-MYB transcription factor homologous to *ARABIDOPSIS TRANSPARENT TESTA 2*, as a casual gene for seed color variation in lettuce through GWAS and fine mapping^44^. The authors demonstrated that knockout of the *LsTT2* gene converted black seeds to white seeds in lettuce, indicating its key role in the seed color regulation. Notably, our GWAS analysis also identified this gene as a candidate for seed color variation, suggesting that the seed color variations among our diverse panel are likely regulated by the same genetic mechanism. This finding supported the hypothesis of a close linkage between seed color and thermoinhibition, or co-localization of their casual loci, and thereby each trait is regulated by distinct genetic pathways rather than a single pleiotropic pathway.

The prevalence of black seeds with reduced thermoinhibition in the lettuce population further suggested that two gene alleles, promoting darker color and low thermoinhibition, respectively, have become linked and fixed together in the lettuce population. This fixation may have resulted from a positive selection, particularly in accessions adapted to environments with intense ultraviolet (UV) radiation, such as hot, sunny climates. Anthocyanins, the pigments abundant in dark-colored seeds, can scavenge reactive oxygen species, thereby limiting oxidative stress during germination under high UV exposure^56^. This protective effect likely enhances survival and successful germination, giving dark seeds with reduced thermoinhibition (thermotolerant germination) a selective advantage in hot summer climates, such as those found in the Mediterranean region. Consequently, both alleles may have become fixed in the lettuce population, contributing to the observed trait distribution.

However, the possibility that these traits are regulated by the same genetic pathway cannot be completely excluded. Our GWAS analysis on seed color variations uncovered additional candidate genomic regions on chromosome 3 and 8, which also appeared to be associated with the thermoinhibition trait. The region on chromosome 8 was located only 860 kb from a thermoinhibition-associated region, while the region on chromosome 3 co-localized with four SNPs displaying near-threshold P-values for thermoinhibition (< 3.7E-06) (Fig. S2). This result suggests potential pleiotropic effects of a shared mechanism on both traits, as the co-localization or nearby positioning of all three chromosomal candidate regions is highly improbable. Definitive determination of whether the correlation between seed color and thermoinhibition is due to pleiotropy or tight linkage warrants further genetic and molecular investigations, such as knockout of the casual genes.

Our germination data indicated that *L. serriola* had a significantly greater germination rate under high temperature conditions compared to cultivated lettuce, suggesting that thermoinhibition is reduced in *L. serriola*. Typically, domestication is associated with the loss of dormancy and a relaxation of environmental controls on germination. However, our data revealed that cultivated lettuce exhibited greater sensitivity to temperature (i.e., greater thermoinhibition) during germination compared to wild *L. serriola* accessions, suggesting an increase in thermoinhibition during the domestication process.

Lettuce is believed to have been domesticated in the Mediterranean regions, which are characterized by hot summers^57^. This evolutionary background suggests that thermoinhibition likely served as an adaptive trait in early *L. sativa* accessions, preventing germination during unsuitably warm periods. On the other hand, since *L. serriola*, a progenitor of cultivated lettuce, displays reduced thermoinhibition, it can be also proposed that *L. serriola* might evolve to weaken thermoinhibition. *L. serriola* is known to be more tolerant to abiotic stresses such as drought, heat, and cold compared to cultivated lettuce^58–60^. The enhanced thermotolerance likely allows this species to further lessen the constraint of thermoinhibition, expanding its geographical range^61^.

Extensive studies on *L. serriola* have identified critical genetic components related to thermoinhibition. Through QTL analysis, one study pinpointed *LsNCED4*, a gene encoding an enzyme involved in ABA synthesis, as a causal gene for reduced thermoinhibition. In *L. serriola*, a mutation in the stop codon of *LsNCED4* resulted in a non-functional protein, leading to lower levels of ABA in heat-exposed seeds, and facilitating germination under heat stress conditions. The presence of this mutated allele in *L. serriola*, likely derived from the wild-type allele, further supports the idea that reduced thermoinhibition in *L. serriola* may be a result of natural selection to adapt to a broader range of climates. A comprehensive survey of alleles among *L. serriola* and cultivated lettuce populations is needed to provide a definitive answer.

Previous studies discovered two causal loci for thermoinhibition variation: *LsNCED4* on chromosome 6^34^, and *LsERF1* on chromosome 9^30^. These studies utilized QTL analysis on biparental population, where genetic variation is inherently limited to two accessions. In contrast, our GWAS analysis, leveraging a genetically diverse panel, identified a region on chromosome 9 near the *LsERF1* locus, potentially implicating the same gene, as well as additional quantitative trait nucleotides (QTNs) on chromosome 4, 5, and 8 (Fig. 5). The discovery of these novel QTNs highlights the complexity of genetic regulation in thermoinhibition and demonstrates the benefits of using diverse germplasm in genetic studies. Overall, our GWAS analysis not only enriches our understanding of the genetic mechanisms underlying thermoinhibition but also provides lettuce breeding efforts with a broader toolkit for developing cultivars with enhanced heat resilience. This is crucial for mitigating the impact of climate change on crop productivity and agricultural sustainability.

## Methods

### Seed production of the GWAS mapping population

A diverse panel of 521 *Lactuca* spp. accessions, including 12 wild lettuce accessions (eleven *L. serriola* and one *L. saligna*) and 509 cultivated lettuce accessions (*L. sativa)* (Table S1), were selected from the USDA-ARS germplasm collections in Salinas, CA. For an initial germination trial, we used seeds harvested between 1977 and 2017, which were stored at -20 °C in closed boxes with silica gel desiccant to control humidity. GWAS were conducted using freshly harvested seeds (May - July 2019) from plants grown in 6.4 cm (2.5 inches) diameter soil pots under natural light conditions in a greenhouse with mean air temperature ranging from 17 to 33 °C.

### Phenotypic analyses

Germination tests were conducted by sowing 25 seeds on Whatman grade 1 filter paper (85 mm diameter) in 100 × 15 mm Petri dishes (Fisher scientific), moistened with 4 mL of deionized water. The petri dishes were arranged in a randomized block design within a germination chamber (Percival GR-41 L), programmed to a 16-hour light/8-hour dark cycle. For germination under high temperature conditions, the chamber temperature was set to 34 °C during the day and 27 °C at night. Conversely, for control germination, the chamber temperature was maintained at 21 °C. Germination success was quantified by the emergence of at least a 2 mm radicle and fully opened cotyledons at six days after imbibition. To minimize positional effects, the petri dishes were rotated every 24 h within the germination chamber. The thermoinhibition rate was calculated as the ratio of seeds germinated under heat stress conditions to those germinated under the control condition (21 °C) (Table S1). This experiment was replicated three times under identical conditions. Statistical analysis of the germination data was conducted using the ‘aov’ functiuon for Analysis of Variance (ANOVA), and the Tukey’s HSD test for pairwise comparison implemented in the R package^62^.

### GWAS analysis

The SNP data for the GWAS analysis was extracted from a public lettuce genotype dataset, (NCBI Project: PRJEB40369)^63^, where the variant SNPs (186,006) were identified based on the lettuce reference genome version 8 ^64^. Briefly explained, missing data within the initial SNP dataset were imputed using Beagle v5^65^, followed by filtering to retain SNPs with a minor allele frequency of at least 5%.

Association mapping analyses for seed thermoinhibition and seed color traits were conducted using the R-package Genome Association and Prediction Integrated Tool (GAPIT) version 3^66^. We employed the Settlement of MLM Under Progressively Exclusive Relationship (SUPER)^67^. Population stratification was corrected by PCA, setting PCA.total to 3, with all other parameters kept at their default settings. Significant associations were determined using a Bonferroni-corrected P-value threshold (0.05 divided by the total number of SNPs), corresponding to 2.69E-7 or − log_10_ (P-value) of 6.57.

### Identification of candidate genes

To identify potential candidate genes, we determined linkage disequilibrium (LD) blocks by calculating pairwise LD values (*r*^2^) between significant SNPs and neighboring SNPs within a 100 kb window, using PLINK version 2^68^. LD blocks were established by merging SNPs with a *r*^2^ value of at least 0.5, indicative of strong LD, which represent genomic regions where SNPs are co-inherited. If the resultant LD block was smaller than 200 kb, the genomic region was extended to up to 200 kb to ensure comprehensive candidate gene identification. These LD blocks were visualized using Haploview 4.2^69^ and candidate genes within the LD blocks were retrieved based on annotations of the lettuce reference genome^64^.

RNA expression levels of the candidate genes under heat stress conditions were obtained from public datasets (NCBI accession GSE241604). Hierarchical clustering analyses were conducted using the hcluster method from the R package amap (version 0.8.16)^70^. The resulting clusters were visualized using the heatmap.2 method from the R package ggplots version 3.1.3^62^.

### Gene ontology (GO) annotation

GO annotation of candidate genes was conducted using the Trinotate pipeline (https://trinotate.github.io/)^71^, and custom PERL scripts. Briefly explained, lettuce protein sequences were subjected to BLASTP searches against UniProtKB/Swiss-Prot database–a manually annotated, non-redundant protein sequence database. GO terms and biological functions were assigned to lettuce genes based on matches in the UniprotKB, with a stringent E-value threshold of less than 1E-20 to ensure high relevance and specificity.

## Electronic supplementary material

Below is the link to the electronic supplementary material.

Supplementary Material 1

Supplementary Material 2

## Data Availability

The lettuce SNP data for the GWAS analysis are available in the NCBI (Project ID: PRJEB40369). RNA-seq data for expression analysis of the candidate genes were obtained from public datasets (NCBI accession GSE241604). All additional relevant data are included in the manuscript and the Supporting Information files.
