# Supplementary material for: Genome-wide association studies in lettuce reveal the interplay of seed age, color, and germination under high temperatures
Source: Sci Rep. 2025 Jan 3;15:733. doi: 10.1038/s41598-024-84197-3 (PMC11698953; doi:10.1038/s41598-024-84197-3)
Supplement: Supplementary file 2 — Supplementary Material 2 [file 41598_2024_84197_MOESM2_ESM.docx]

**Genome-wide association studies for seed thermoinhibition in lettuce reveal the interplay of seed age, color, and germination under high temperatures**

**Sookyung Oh^1^, Ezekiel Ahn^1^, Ainong Shi^2^, Beiquan Mou^3*^ and Sunchung Park^1*^**

^1^U.S. Department of Agriculture, Agricultural Research Service, Beltsville, MD 20705, USA

^2^Horticulture Dept., University of Arkansas, Fayetteville, AR 72701, USA

^3^U.S. Department of Agriculture, Agricultural Research Service, Salinas, CA 93905, USA

**Supplementary information**

Supplementary Figure S1

Supplementary Figure S2

Supplementary Table S1-9


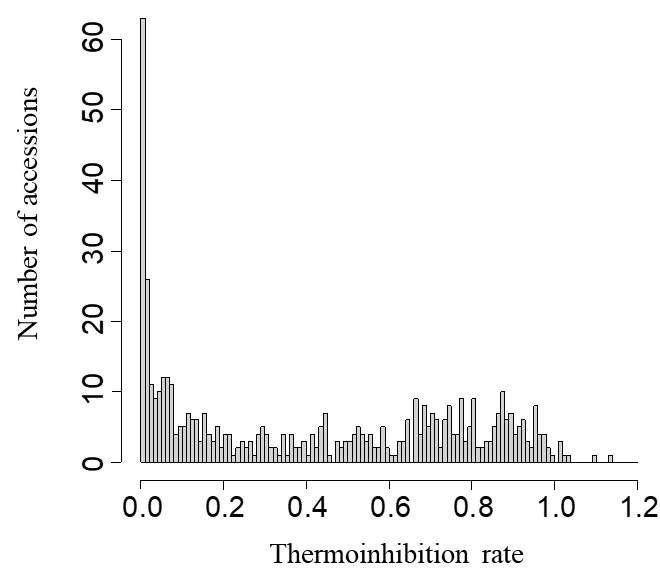


**Figure S1. Distribution of accessions by thermoinhibition rate.** This histogram illustrates the thermoinhibition rate represented by a ratio of seeds germinated under high-temperature conditions (34°C) to those germinated under control conditions (21°C), highlighting the variance in thermoinhibition among assessed accessions.


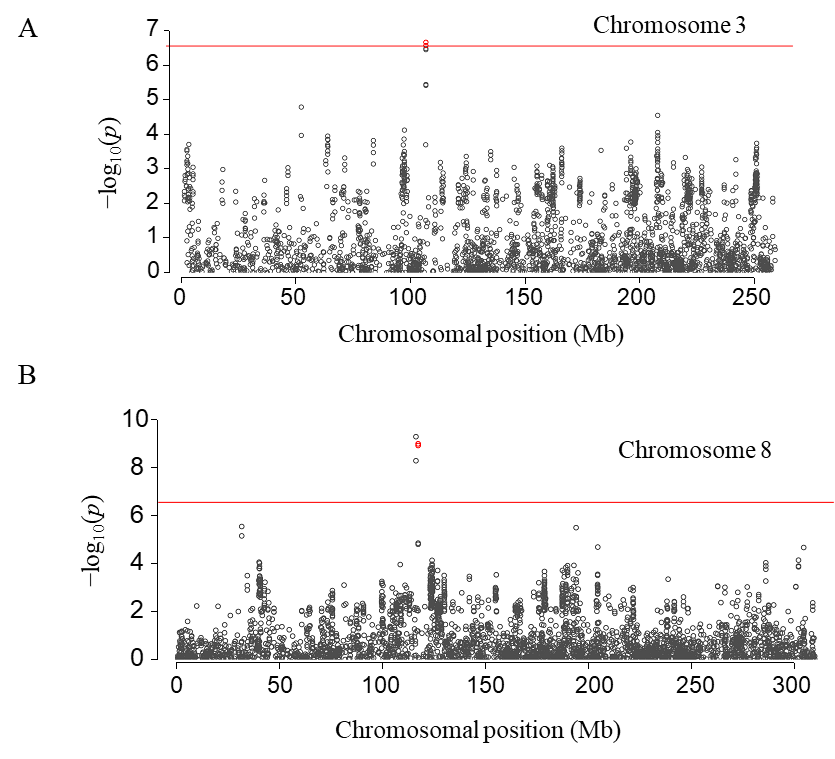


**Figure S2. Manhattan plots of chromosome 3 (A) and 8 (B) showing significant SNPs for seed color and thermoinhibition.** Manhattan plots display significant SNPs for seed color (red circles) and thermoinhibition (black circles). The red lines represent the genome-wide significance threshold (P-value <2.7E-7, −log10(p) = 6.57)

**Supplementary Tables**

Table S1. *Lactuca* accessions and germination phenotypes for GWAS

Table S2. ANOVA test of heat germination across seven harvest year groups

Table S3 ANOVA test of horticultural type effects on seed thermoinhibition

Table S4. ANOVA of seed color effects on seed thermoinhibition

Table S5. Significant SNP associated with thermoinhibition rate in lettuce

Table S6. GWAS LD blocks and Causal genes previously identified for Thermoinhibition or Seed color

Table S7. Candidate genes associated with thermoinhibition and their GO annotations

Table S8. Significant SNP associated with seed color

Table S9. Candidate genes associated with Seed Color and their GO annotations
